# Cognitive and Psychological Mediators of the Social Gradient in Tobacco Use Initiation Among Adolescents: Evidence from the ABCD Study

**DOI:** 10.31586/jbls.2024.1035

**Published:** 2024-09-04

**Authors:** Shervin Assari, Hossein Zare

**Affiliations:** 1 Department of Internal Medicine, Charles R. Drew University of Medicine and Science, Los Angeles, CA, United States; 2 Department of Family Medicine, Charles R. Drew University of Medicine and Science, Los Angeles, CA, United States; 3 Department of Urban Public Health, Charles R. Drew University of Medicine and Science, Los Angeles, CA, United States; 4 Marginalization-Related Diminished Returns (MDRs) Center, Los Angeles, CA, United States; 5 Department of Health Policy and Management, Johns Hopkins Bloomberg School of Public Health, Baltimore, MD, United States; 6 School of Business, University of Maryland Global Campus (UMGC), Adelphi, MD, United States

**Keywords:** Tobacco use, Adolescents, Social gradients, Substance use harm knowledge, Tobacco susceptibility, ABCD study, Health disparities, Socioeconomic status

## Abstract

**Background:**

Tobacco use among adolescents is a significant public health concern, with early initiation leading to long-term health risks. Understanding the factors that contribute to the initiation of tobacco use is crucial for developing effective prevention strategies. This study investigates the roles of substance use harm knowledge and tobacco susceptibility in mediating the relationship between social gradients (race, ethnicity, and socioeconomic status) and tobacco use initiation among adolescents.

**Methods:**

Data from the Adolescent Brain Cognitive Development (ABCD) study, comprising a racially, ethnically, and economically diverse sample of tobacco-naive adolescents aged 9 to 16, were analyzed. Structural equation modeling (SEM) was used to test whether substance use harm knowledge and tobacco susceptibility mediate the effects of socioeconomic status (SES) on the initiation of tobacco use.

**Results:**

Findings indicated that both substance use harm knowledge and tobacco susceptibility partially mediate the relationship between SES and tobacco use initiation. Adolescents from lower SES backgrounds exhibited lower levels of harm knowledge and higher levels of tobacco susceptibility, which increased their likelihood of initiating tobacco use.

**Conclusion:**

This study highlights the complex interplay between social determinants and individual cognitive and psychological factors in influencing tobacco use initiation among adolescents. Public health interventions that enhance harm knowledge and reduce susceptibility to tobacco use are crucial for preventing initiation, particularly among racially, ethnically, and economically diverse adolescents. These efforts can help reduce health disparities and promote health equity.

## 1. Introduction

Tobacco use among adolescents remains a significant public health concern [1], with early initiation often leading to prolonged use and increased risk of addiction and adverse health outcomes [2]. Understanding the factors that contribute to the initiation of tobacco use during adolescence is crucial for developing effective prevention strategies. The Adolescent Brain Cognitive Development (ABCD) study [3] provides a unique opportunity to explore these factors in a diverse sample of adolescents followed longitudinally from age 9 to 16. This study leverages the ABCD study data to examine how cognitive and psychological elements influence the initiation of tobacco use among racially, ethnically, and economically diverse adolescents.

Racial, ethnic, and economic disparities in tobacco use have been well-documented [4], with minority and low-income adolescents often exhibiting higher rates of initiation and use compared to their White and higher-income counterparts [4, 5]. These disparities can be attributed to various social determinants of health, including differential access to resources, exposure to tobacco marketing, and varying levels of parental and community support [6]. Previous research has highlighted the social gradient in health behaviors, where individuals from lower socioeconomic backgrounds are more likely to engage in risk behaviors, including tobacco use [7]. However, the mechanisms through which these social gradients influence tobacco use initiation remain underexplored.

Two critical cognitive and psychological factors that may mediate the relationship between social gradients and tobacco use initiation are substance use harm knowledge [8] and tobacco susceptibility [9]. Substance use harm knowledge refers to an individual’s awareness and understanding of the negative consequences associated with tobacco use [10]. Adolescents with higher levels of harm knowledge are less likely to initiate tobacco use, as they are more aware of its risks [11]. Tobacco susceptibility encompasses curiosity, openness to use, and the perceived likelihood of future use [9, 12]. Adolescents who are more susceptible to tobacco use are more likely to experiment with and eventually initiate tobacco use [9]. Theoretical frameworks suggest that social factors such as race, ethnicity, and socioeconomic status can influence these cognitive and psychological elements, thereby affecting the likelihood of tobacco use initiation.

## 2. Objectives and Hypotheses

The primary objective of this study is to investigate the mediating roles of substance use harm knowledge and tobacco susceptibility in the relationship between social gradients (race, ethnicity, and socioeconomic status) and the initiation of tobacco use among adolescents. We hypothesize that social gradients will predict the initiation of tobacco use, with minority and low-income adolescents being more likely to start using tobacco. Additionally, we hypothesize that substance use harm knowledge will mediate the relationship between social gradients and tobacco use initiation, such that adolescents from lower socioeconomic backgrounds will have lower harm knowledge, increasing their likelihood of initiating tobacco use. Finally, we hypothesize that tobacco susceptibility will also mediate this relationship, with higher susceptibility among minority and low-income adolescents leading to higher rates of tobacco use initiation. This study aims to shed light on the cognitive and psychological mechanisms that underlie the social gradients in tobacco use initiation among adolescents. By identifying the mediating roles of substance use harm knowledge and tobacco susceptibility, we can better understand the pathways through which social factors influence tobacco use behaviors. The findings of this study have significant implications for public health interventions, highlighting the need to address cognitive and psychological factors in prevention programs. By targeting these mediators, interventions can be more effective in preventing the initiation of tobacco use, particularly among racially, ethnically, and economically diverse adolescents, ultimately contributing to the reduction of health disparities and promoting health equity.

## 3. Methods

### 3.1. Design and Setting:

This study utilized data from the Adolescent Brain Cognitive Development (ABCD) Study, a large, longitudinal cohort study designed to assess brain development and health in adolescents across the United States. The baseline data collection began between 2016 and 2018, and the study is ongoing.

### 3.2. Sample and Sampling:

The ABCD Study includes a racially, ethnically, and socioeconomically diverse sample of adolescents recruited from 21 research sites nationwide. While the sample is not nationally representative, it closely reflects the diversity of the U.S. population. Additionally, participants were predominantly enrolled through schools.

### 3.3. Analytical Sample:

For this analysis, we included tobacco-naive adolescents aged 9 to 16 years at baseline, who had complete data on the key variables of interest, resulting in a final sample size that captures the diverse socioeconomic and demographic characteristics of the population.

### 3.4. Measures:

#### Predictors

##### Socioeconomic Status (SES):

SES was assessed using a composite score that included parental education, household income, family structure, and financial difficulties. This composite measure was used to capture the multidimensional nature of socioeconomic status in the participants’ households.

#### Outcome

##### Tobacco Use Initiation:

Tobacco use initiation was defined as the first reported use of any tobacco product, including cigarettes, e-cigarettes, and other tobacco-related products, at any follow-up assessment during the study period.

#### Mediators

The proposed model included substance use harm knowledge and tobacco susceptibility as mediators. These mediators were hypothesized to transmit the effects of SES on future tobacco initiation, suggesting that they would be associated with both SES and subsequent tobacco initiation.

##### Substance Use Harm Knowledge:

Substance use harm knowledge was measured using a standardized questionnaire assessing adolescents’ understanding of the health risks associated with tobacco and other substance use. Higher scores indicated greater knowledge of the harms associated with substance use.

##### Tobacco Susceptibility:

Tobacco susceptibility was assessed using items from a validated questionnaire that measures openness to future tobacco use, curiosity about tobacco products, and lack of a firm commitment to abstain from tobacco use. Higher scores indicated greater susceptibility to tobacco use.

#### Covariates

All models controlled for potential confounders, including age, sex, and race/ethnicity, which could influence both the mediators and the outcome.

##### Race/Ethnicity:

Race/ethnicity was self-reported by the parents or their guardians and categorized into the following groups: Non-Latino White, Non-Latino Black, Latino, Asian, and Other. These categories were used to examine the social gradient in tobacco use initiation.

##### Age:

Age at baseline was categorized as a dichotomous variable with two groups: 10 years and 9 years (reference category). Age was calculated based on the interval between the participant’s date of birth and the date of enrollment.

##### Sex:

Sex was recorded as a dichotomous variable, coded as 1 for male and 0 for female.

### 3.5. Statistical Analysis:

Data analysis was conducted using Stata 18.0. Structural Equation Modeling (SEM) was employed to test the hypothesized mediation model, examining whether substance use harm knowledge and tobacco susceptibility mediated the effects of SES on tobacco use initiation. SEM allowed for the simultaneous evaluation of both direct and indirect effects of SES and race/ethnicity on tobacco use initiation through cognitive (substance harm knowledge) and psychological (tobacco susceptibility) mediators. SEM is particularly suited for testing mediations and indirect effects because it enables the examination of complex relationships among multiple variables, including both direct and indirect pathways, within a single model. It allows researchers to specify and test theoretical models that incorporate mediators, offering a clear framework for understanding how independent variables influence dependent variables through intermediary factors. Unlike traditional regression methods, SEM accounts for measurement errors in observed variables, enhancing the accuracy and reliability of results. Additionally, SEM supports the testing of multiple mediators simultaneously, providing a comprehensive view of the mechanisms underlying associations between variables. SEM also offers fit indices that help evaluate the model’s alignment with the observed data, ensuring robust and theoretically sound findings. Model fit was assessed using standard fit indices, including the Comparative Fit Index (CFI), the Tucker-Lewis Index (TLI), and the Root Mean Square Error of Approximation (RMSEA). P-values less than 0.05 were considered statistically significant. We reported standardized beta coefficients, standard errors (SE), their 95% Confidence Intervals (CI), and p-values to present the results comprehensively.

### 3.6. Ethical Considerations:

The ABCD Study was conducted in accordance with ethical standards and approved by institutional review boards at each participating research site. Informed consent was obtained from the parents or legal guardians of the participants, and assent was obtained from the adolescents themselves.

## 4. Results

Table 1 presents the descriptive statistics for the study sample. The sample included 11,815 participants, with the majority identifying as non-Latino White (52.01%), followed by non-Latino Latino (20.35%), non-Latino Black (14.97%), Other (10.42%), and Asian (2.14%). The sex distribution was nearly balanced, with 52.00% male and 48.00% female participants. Regarding the marital status of the household, 67.49% were from married households, while 32.51% were from households where the parents were not married.

The age distribution of the participants was relatively even, with 52.16% aged 9 years and 47.84% aged 10 years. The average years of parental education was 17.164 years (SE = 0.047). The mean household income score was 7.700 (SE = 0.040; range 1–10). Substance use harm knowledge among participants had a mean score of 26.244 (SE = 0.119). The mean tobacco susceptibility score was 1.081 (SE = 0.004), and the subsequent tobacco use mean score was 0.080 (SE = 0.005).

Table 2 summarizes the unadjusted correlations between study variables. Socioeconomic status (SES) was strongly positively correlated with parental education (r = 0.76, p < 0.05) and household income (r = 0.89, p < 0.05). It also had positive correlations with substance use harm knowledge (r = 0.06, p < 0.05) but showed negative correlations with tobacco susceptibility (r = −0.02, p < 0.05) and subsequent tobacco use (r = −0.06, p < 0.05). Parental education was positively correlated with household income (r = 0.62, p < 0.05) and substance use harm knowledge (r = 0.06, p < 0.05), while it was negatively correlated with subsequent tobacco use (r = −0.04, p < 0.05). Household income was positively correlated with substance use harm knowledge (r = 0.02, p < 0.05) but negatively correlated with tobacco susceptibility (r = −0.02, p < 0.05) and subsequent tobacco use (r = −0.04, p < 0.05). Substance use harm knowledge had a significant negative correlation with tobacco susceptibility (r = −0.09, p < 0.05) and subsequent tobacco use (r = −0.09, p < 0.05). Tobacco susceptibility was positively correlated with subsequent tobacco use (r = 0.05, p < 0.05).

**Figure 1.**
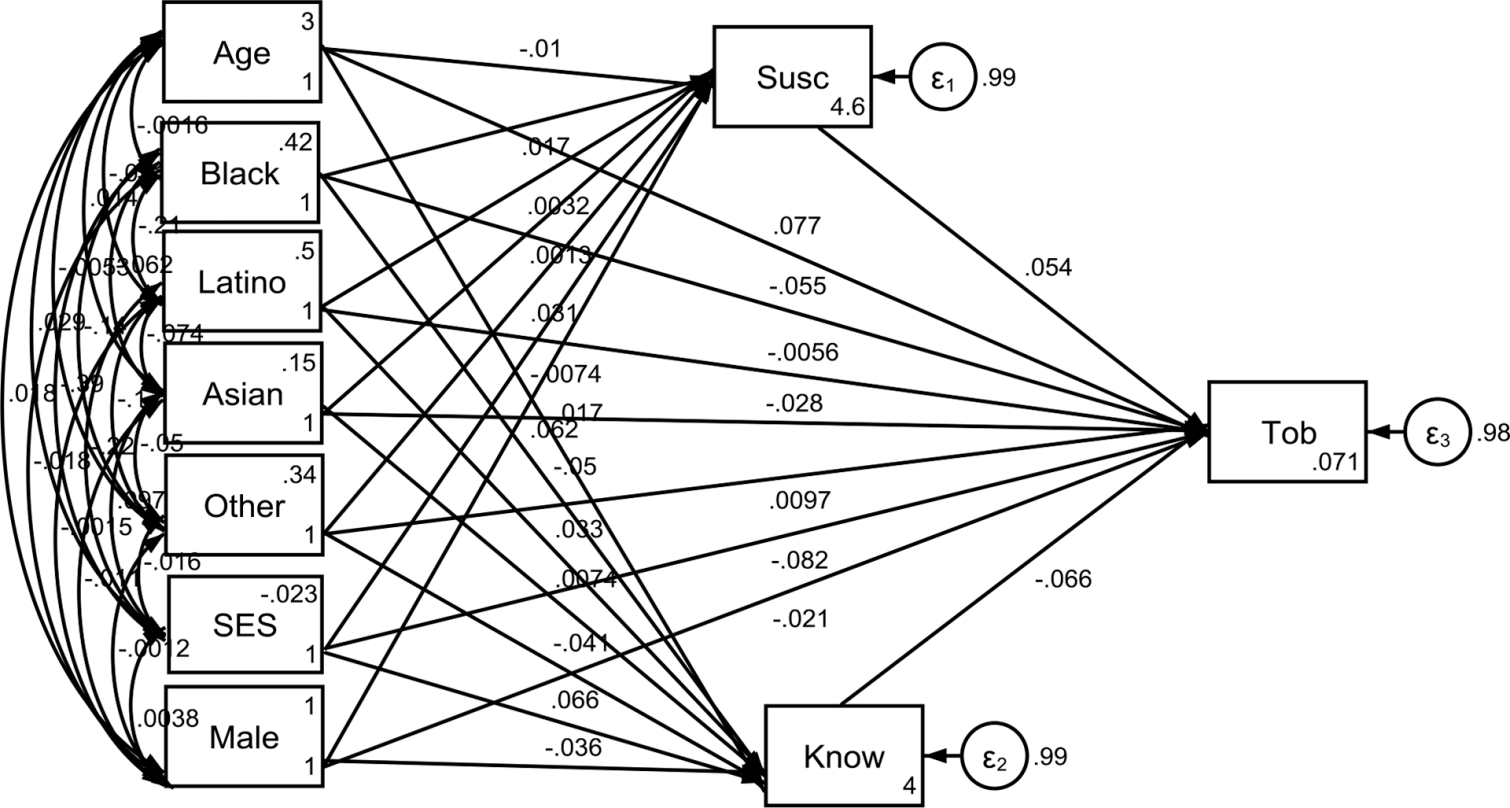
Summary of Structural Equation Model (SEM)

Figure 1 summarizes the SEM results. Findings indicated that both substance use harm knowledge and tobacco susceptibility partially mediate the relationship between SES and tobacco use initiation. Adolescents from lower SES backgrounds exhibited lower levels of harm knowledge and higher levels of tobacco susceptibility, which increased their likelihood of initiating tobacco use.

Table 3 provides the significance of each path in the Structural Equation Model (SEM) examining factors associated with tobacco susceptibility, substance use harm knowledge, and subsequent tobacco use. This model also tested the indirect effect of socioeconomic status (SES) on subsequent tobacco use through tobacco susceptibility and substance use harm knowledge.

SES was not significantly associated with tobacco susceptibility (B = −0.01, SE = 0.01, 95% CI: −0.03 to 0.02, p = 0.544). Among racial and ethnic groups, only individuals identifying as “Other” showed a significant positive association with tobacco susceptibility (B = 0.03, SE = 0.01, 95% CI: 0.01 to 0.05, p = 0.002). The race/ethnicity categories of Black (B = 0.02, SE = 0.01, 95% CI: −0.01 to 0.04, p = 0.158), Latino (B = 0.00, SE = 0.01, 95% CI: −0.02 to 0.02, p = 0.772), and Asian (B = 0.00, SE = 0.01, 95% CI: −0.02 to 0.02, p = 0.891) were not significantly associated with tobacco susceptibility. Age was not significantly associated with tobacco susceptibility (B = −0.01, SE = 0.01, 95% CI: −0.03 to 0.01, p = 0.288), whereas male sex was significantly associated with higher tobacco susceptibility (B = 0.06, SE = 0.01, 95% CI: 0.04 to 0.08, p < 0.001).

SES was positively associated with substance use harm knowledge (B = 0.07, SE = 0.02, 95% CI: 0.02 to 0.11, p = 0.008). Black race/ethnicity was negatively associated with substance use harm knowledge (B = −0.05, SE = 0.03, 95% CI: −0.10 to 0.00, p = 0.050), and the “Other” race/ethnicity category also showed a negative association (B = −0.04, SE = 0.02, 95% CI: −0.08 to 0.00, p = 0.029). The race/ethnicity categories of Latino (B = 0.03, SE = 0.02, 95% CI: −0.01 to 0.07, p = 0.120) and Asian (B = 0.01, SE = 0.02, 95% CI: −0.03 to 0.04, p = 0.665) were not significantly associated with substance use harm knowledge. Age was not significantly associated with substance use harm knowledge (B = 0.02, SE = 0.02, 95% CI: −0.02 to 0.05, p = 0.370), and male sex was marginally negatively associated (B = −0.04, SE = 0.02, 95% CI: −0.07 to 0.00, p = 0.051). The intercept was significant (B = 4.05, SE = 0.09, 95% CI: 3.88 to 4.21, p < 0.001).

Regarding subsequent tobacco use, tobacco susceptibility showed a significant positive association (B = 0.05, SE = 0.01, 95% CI: 0.03 to 0.07, p < 0.001), and substance use harm knowledge had a significant negative association (B = −0.07, SE = 0.01, 95% CI: −0.10 to −0.04, p < 0.001). SES was directly and negatively associated with subsequent tobacco use (B = −0.08, SE = 0.01, 95% CI: −0.10 to −0.06, p < 0.001), independent of tobacco susceptibility and substance use harm knowledge. Black race/ethnicity was significantly negatively associated with subsequent tobacco use (B = −0.05, SE = 0.01, 95% CI: −0.08 to −0.03, p < 0.001), as was Asian race/ethnicity (B = −0.03, SE = 0.01, 95% CI: −0.05 to −0.01, p = 0.003). The race/ethnicity categories of Latino (B = −0.01, SE = 0.01, 95% CI: −0.03 to 0.01, p = 0.595) and “Other” (B = 0.01, SE = 0.01, 95% CI: −0.01 to 0.03, p = 0.315) were not significantly associated. Age was positively associated with subsequent tobacco use (B = 0.08, SE = 0.01, 95% CI: 0.06 to 0.10, p < 0.001), and male sex was negatively associated (B = −0.02, SE = 0.01, 95% CI: −0.04 to 0.00, p = 0.024).

Overall, the model suggests that tobacco susceptibility and substance use harm knowledge partially mediate the effects of SES on subsequent tobacco use. However, SES also has a direct negative effect on subsequent tobacco use that is independent of these mediators.

## 5. Discussion

This study aimed to investigate the mediating roles of substance use harm knowledge and tobacco susceptibility in the relationship between social gradients (race, ethnicity, and socioeconomic status) and the initiation of tobacco use among adolescents. Our findings confirm that both cognitive and psychological elements partially mediate the effects of SES on the future use of tobacco among adolescents. Specifically, adolescents from lower SES backgrounds were more likely to initiate tobacco use, and this relationship was significantly mediated by lower levels of substance use harm knowledge and higher levels of tobacco susceptibility. These results underscore the complex interplay between social determinants and individual cognitive and psychological factors in influencing health behaviors among adolescents.

Our analysis revealed that substance use harm knowledge mediates the relationship between social gradients and tobacco use initiation. Adolescents from lower SES backgrounds tend to have lower levels of harm knowledge, which increases their likelihood of initiating tobacco use. This finding suggests that educational interventions aimed at increasing harm knowledge could be particularly beneficial for adolescents from disadvantaged backgrounds.

Additionally, tobacco susceptibility was found to mediate the relationship between social gradients and tobacco use initiation. Adolescents who are more curious, open to using tobacco, and perceive a higher likelihood of future use are more likely to initiate tobacco use, and these susceptibilities are more prevalent among minority and low-income adolescents. This indicates that efforts to reduce tobacco susceptibility through targeted interventions could help mitigate the risk of tobacco initiation in these populations.

Our findings align with previous research that has documented the social gradient in health behaviors, including tobacco use. Studies have consistently shown that adolescents from lower SES backgrounds and minority groups are at higher risk of initiating tobacco use. However, this study extends the existing literature by elucidating the mediating roles of cognitive and psychological factors. Previous research has primarily focused on direct associations between social determinants and health behaviors, often overlooking the underlying mechanisms. By highlighting the roles of substance use harm knowledge and tobacco susceptibility, this study provides a more nuanced understanding of how social gradients influence tobacco use initiation. This contribution is significant as it underscores the importance of addressing both social and individual factors in prevention strategies.

### 5.1. Implications

The findings of this study have important implications for public health interventions aimed at preventing tobacco use among adolescents. First, increasing substance use harm knowledge through educational programs could be a key strategy in reducing tobacco use initiation, particularly among adolescents from lower SES backgrounds. Schools and community organizations can play a crucial role in disseminating information about the risks of tobacco use, tailored to the specific needs of disadvantaged populations. Second, interventions targeting tobacco susceptibility are essential. Programs that reduce curiosity and openness to tobacco use, and diminish the perceived likelihood of future use, can help lower the risk of initiation. These interventions could include counter-marketing campaigns, parental and peer education, and activities that promote healthy, tobacco-free lifestyles.

### 5.2. Limitations and Future Research

While this study provides valuable insights, it also has several limitations. The reliance on self-reported data may introduce bias, as adolescents may underreport or overreport their tobacco use and susceptibility. Future research could benefit from incorporating objective measures of tobacco use and susceptibility. Additionally, the study focuses on a specific age range (9 to 16 years), and the findings may not be generalizable to older adolescents or young adults. Longitudinal studies that follow individuals beyond adolescence could provide a more comprehensive understanding of the long-term effects of cognitive and psychological mediators on tobacco use. Furthermore, while this study highlights the mediating roles of harm knowledge and susceptibility, other potential mediators, such as peer influence, parental smoking, and neighborhood factors, should also be explored in future research to provide a more holistic understanding of the pathways to tobacco use initiation.

## 6. Conclusion

In conclusion, this study underscores the importance of cognitive and psychological factors in mediating the relationship between social gradients and the initiation of tobacco use among adolescents. By identifying substance use harm knowledge and tobacco susceptibility as key mediators, the findings highlight the need for multifaceted prevention strategies that address both social determinants and individual factors. Public health interventions that enhance harm knowledge and reduce susceptibility to tobacco use are crucial for preventing tobacco initiation, particularly among racially, ethnically, and economically diverse adolescents. These efforts can contribute to reducing health disparities and promoting health equity, ultimately leading to healthier outcomes for all adolescents.

## Figures and Tables

**Table 1.** Descriptive Statistics

	n	%
**Race/Ethnicity**		
Non-Latino White	6,101	52.01
Non-Latino Black	1,756	14.97
Latino	2,387	20.35
Asian	251	2.14
Other	1,222	10.42
**Sex**		
Female	5,628	48.00
Male	6,098	52.00
**Marital Status of the Household**		
Not Married	3,813	32.51
Married	7,916	67.49
**Age**		
9 Yrs	6,128	52.16
10 Yrs	5,621	47.84
	Mean	SE
**Socioeconomic Status (SES)**	0.292	0.025
**Parent Education (Years)**	17.164	0.047
**Household Income**	7.700	0.040
**Substance Use Harm Knowledge**	26.244	0.119
**Tobacco Susceptibility**	1.081	0.004
**Tobacco Use (Subsequent)**	0.080	0.005

**Table 2.** Bivariate Correlations

	1	2	3	4	5	6	7	8	9	10	11	12	13
1 Age (10 Yrs)	1.00												
2 Male	0.02*	1.00											
3 Married Household	0.01	0.02*	1.00										
4 Race/Ethnicity (Black)	0.00	−0.02*	−0.35*	1.00									
5 Race/Ethnicity (Latino)	−0.02*	0.00	−0.11*	−0.21*	1.00								
6 Race/Ethnicity (Asian)	0.01	−0.01*	0.07*	−0.06*	−0.07*	1.00							
7 Race/Ethnicity (Other)	−0.01	0.00	−0.02*	−0.14*	−0.17*	−0.05*	1.00						
8 Socioeconomic Status (SES)	0.03*	0.01	0.72*	−0.39*	−0.23*	0.09*	−0.02*	1.00					
9 Parental Education	0.02*	0.01	0.35*	−0.22*	−0.30*	0.10*	0.02*	0.76*	1.00				
10 Household Income	0.04*	0.01	0.55*	−0.37*	−0.22*	0.06*	−0.02*	0.89*	0.62*	1.00			
11 Substance Use Harm Knowledge	0.02*	−0.03*	0.07*	−0.05*	0.03*	0.02*	−0.05*	0.06*	0.06*	0.02*	1.00		
12 Tobacco Susceptibility	−0.01	0.06*	−0.01	0.01	0.00	0.00	0.03*	−0.02*	0.00	−0.02*	−0.09*	1.00	
13 Tobacco Use (Subsequent)	0.07*	−0.02*	−0.06*	−0.02*	0.02*	−0.03*	0.02*	−0.06*	−0.04*	−0.04*	−0.09*	0.05*	1.00

* p < 0.05

**Table 3.** Summary of Structural Equation Model (SEM)

	B	SE	95%	CI	p
Tobacco Susceptibility					
Socioeconomic Status (SES)	−0.01	0.01	−0.03	0.02	0.544
Race/Ethnicity (Black)	0.02	0.01	−0.01	0.04	0.158
Race/Ethnicity (Latino)	0.00	0.01	−0.02	0.02	0.772
Race/Ethnicity (Asian)	0.00	0.01	−0.02	0.02	0.891
Race/Ethnicity (Other)	0.03	0.01	0.01	0.05	0.002
Age (10 Yrs)	−0.01	0.01	−0.03	0.01	0.288
Sex (Male)	0.06	0.01	0.04	0.08	< 0.001
Intercept	4.63	0.05	4.54	4.72	< 0.001
Substance Use Harm Knowledge					
Socioeconomic Status (SES)	0.07	0.02	0.02	0.11	0.008
Race/Ethnicity (Black)	−0.05	0.03	−0.10	0.00	0.050
Race/Ethnicity (Latino)	0.03	0.02	−0.01	0.07	0.120
Race/Ethnicity (Asian)	0.01	0.02	−0.03	0.04	0.665
Race/Ethnicity (Other)	−0.04	0.02	−0.08	0.00	0.029
Age (10 Yrs)	0.02	0.02	−0.02	0.05	0.370
Sex (Male)	−0.04	0.02	−0.07	0.00	0.051
Intercept	4.05	0.09	3.88	4.21	< 0.001
Tobacco Use (Subsequent)					
Tobacco Susceptibility	0.05	0.01	0.03	0.07	< 0.001
Substance Use Harm Knowledge	−0.07	0.01	−0.10	−0.04	< 0.001
Socioeconomic Status (SES)	−0.08	0.01	−0.10	−0.06	< 0.001
Race/Ethnicity (Black)	−0.05	0.01	−0.08	−0.03	< 0.001
Race/Ethnicity (Latino)	−0.01	0.01	−0.03	0.01	0.595
Race/Ethnicity (Asian)	−0.03	0.01	−0.05	−0.01	0.003
Race/Ethnicity (Other)	0.01	0.01	−0.01	0.03	0.315
Age (10 Yrs)	0.08	0.01	0.06	0.10	< 0.001
Sex (Male)	−0.02	0.01	−0.04	0.00	0.024
Intercept	0.07	0.08	−0.09	0.23	0.390
